# Preliminary exploration of clinical factors affecting acute toxicity and quality of life after carbon ion therapy for prostate cancer

**DOI:** 10.1186/s13014-019-1303-3

**Published:** 2019-06-04

**Authors:** Yafang Zhang, Ping Li, Qi Yu, Shuang Wu, Xue Chen, Qing Zhang, Shen Fu

**Affiliations:** 1 Department of Radiation Oncology, Shanghai Proton and Heavy lon Center, Fudan University Cancer Hospital, No.4365 Kang Xin Road, Shanghai, 201321 China; 2 Department of Radiation Oncology, Shanghai Proton and Heavy lon Center, Shanghai, 201321 China; 3 Shanghai Engineering Research Center of Proton and Heavy lon Radiation Therapy, Shanghai, 201321 China; 4 Department of Radiation Oncology, Fudan University Cancer Hospital, Shanghai, 200020 China; 5 Key Laboratory of Nuclear Physics and lon-Beam Application (MOE), Fudan University, Shanghai, 200433 China; 6 Department of Radiation Oncology, Shanghai Concord Cancer Hospital, Shanghai, 200020 China

**Keywords:** Prostate cancer, Carbon ion therapy, Quality of life, GU toxicity

## Abstract

**Purpose:**

To assess toxicity and quality-of-life (QOL) after carbon ion radiotherapy (CIRT) at the Shanghai Proton and Heavy Ion Center (SPHIC) and identify clinical factors that correlate with urinary, bowel and sexual function.

**Methods:**

Sixty-four patients with localized prostate cancer admitted from July 2015 to January 2018 underwent CIRT. At baseline and 5 time-points after radiotherapy, we assessed patients’ QOL using the 26-item edition of the Expanded Prostate Cancer Index-Composite (EPIC-26) Chinese version. Logistic regression was performed to identify clinical factors associated with acute genitourinary (GU) toxicity and relative QOL.

**Results:**

By the end of CIRT, urinary irritation/obstruction temporarily declined (− 7.92 ± 1.76, *p* < .001). For urinary incontinence, bowel and sexual QOL, the scores remained stable at 2-year follow-up. The occurrences of acute Grade 1 and 2 GU toxicity were 20.3 and 10.9%, respectively, and of late Grade 1 and 2 GU toxicity were 3.1 and 1.6%, respectively. No acute or late gastrointestinal (GI) toxicity occurred. Transurethral resection of the prostate (TURP) was a risk factor that predicted a decline in urinary related QOL, and age made a difference to bowel-related QOL. For sexual QOL, castration status was a remarkable risk factor. An international prostate symptom score (IPSS) ≥8 increased the risk of Grade 1–2 acute GU toxicity 5.3-fold.

**Conclusion:**

Patients with prostate cancer had favorable QOL after CIRT. IPSS ≥8 was a risk factor to acute GU toxicity, and TURP predicted a decline in urinary QOL. Age was related to bowel QOL, and castration status was associated with sexual QOL.

**Trial registration:**

Carbon Ion Radiotherapy for the Treatment of Localized Prostate Cancer, NCT02739659. Registered April 15, 2016.

## Background

Prostate cancer is the most common urologic cancer among Chinese men with largest increase in incidence of all cancers in 2015 [1]. Carbon ion radiotherapy (CIRT) have high relative biological effectiveness and a low oxygen enhancement ratio, resulting in stronger cytocidal effects than conventional radiotherapy [2, 3]. Further, carbon ions offer the advantage of obvious maximum dose deposition in the deep localized region, the so-called Bragg peak, which can lead to more accurate dose concentration to the tumor, as well as reduced toxicity to the surrounding healthy tissues [4]. Therefore, CIRT could be an ideal treatment strategy for clinically localized prostate cancer.

Some clinical trials have reported excellent disease control and favorable toxicity of CIRT for prostate cancer [5–8]. However, there are few studies on the quality of life (QOL) and toxicity after CIRT for prostate cancer [5, 9], and none has explored the factors associated with QOL changes after CIRT. Identifying the factors affecting toxicity and QOL can characterize the patients who will benefit the most from CIRT. Phase I/II clinical trials (NCT02739659) of CIRT for localized prostate cancer at the Shanghai Proton and Heavy Ion Center (SPHIC) were initiated in 2016, using the IONTRIS particle therapy system. The purpose of this article was to demonstrate the QOL change from CIRT for prostate cancer at the SPHIC and investigate the factors that affected acute toxicity and long-term QOL changes.

## Methods

### Participants and treatments

A total of 102 patients with prostate cancer were consecutively admitted from July 2015 to January 2018. The inclusion criteria were men with pathologically confirmed localized prostate cancer who received CIRT as the radical treatment. Patients with visceral or bone metastases [10], pelvic nodes metastases [11], postoperative radiotherapy [4], and recurrence after either surgery or radiotherapy [7] were excluded. Finally, 64 eligible patients participated in this study. Risk category was defined using the National Comprehensive Cancer Network (NCCN) prostate cancer guidelines [12]. This research had been approved by the institutional review boards of the SPHIC, and all patients provided written informed consent before admission.

Before CT simulation and every irradiation, patients were required to empty the rectum and fill the bladder. Patients are instructed to drink 330 ml of water 30–60 min prior to simulation and daily treatment. The organ at risk, including the rectum and bladder, was contoured on the basis of the Male Radiation Therapy Oncology Group (RTOG) Normal Pelvis Atlas [11]. The clinical target volume (CTV) was defined as the prostate alone for low-risk prostate cancer, prostate plus proximal 1–2.5 cm of seminal vesicles for intermediate- to very high-risk localized prostate cancer. The planning target volume was calculated from the CTV using the lateral margins of 10 mm as well as an anterior and posterior margin of 5 mm. Pelvic node radiation therapy was not used. Patients enrolled before January 2016 were treated by 66 photon Gray equivalent (GyE) (physical carbon ion dose [Gy] × RBE, which was assigned to be 3.0 at the distal part of the spread out Bragg peak) in 24 fractions, and by 59.2–60.8 GyE in 16 fractions thereafter. The CIRT was delivered once a day, 5 days a week.

Hormonal therapy was not administrated to low-risk patients, while intermediate-risk patients recommended to receive at least 6 months of hormonal therapy. And high-risk and very high-risk patients recommended for at least 2 years hormonal therapy.

### Pretreatment assessment and follow-up

Before CIRT initiation, demographic characteristics, international prostate symptom score (IPSS) and past medical history including transurethral resection of prostate (TURP) and hemorrhoids was recorded. Given the IPSS symptom questions were responsive to lower urinary tract symptoms [13], we divided the patients into 2 groups: IPSS ≤7 (mild symptoms) and IPSS ≥8 (moderate/severe symptoms), as previously described [14, 15].

The urinary-related adverse events, such as obstruction and cystitis, were defined as genitourinary (GU) toxicity, whereas the bowel-related adverse events were defined as gastrointestinal (GI) toxicity. Acute toxicity was evaluated using the Common Toxicity Criteria Adverse Event 4.03. Late toxicity was assessed by the RTOG/European Organisation for Research and Treatment of Cancer (EORTC) Late Radiation Morbidity scoring scheme [16].

The Expanded Prostate Cancer Index-Composite (EPIC) is a well-established patient-reported outcome questionnaire to monitor QOL among prostate cancer survivors. The 26-item version of EPIC, also known as the EPIC-26, contains symptom-based domains, such as urinary incontinence, urinary irritation/obstruction, sexual and bowel. The EPIC-26 domains, scoring from 0 (poorest) to 100 (best), can be tracked over time to understand symptom burden, functional outcomes and the impact of adverse effects [10]. The EPIC-26 Chinese version [17] was used in our study.

The QOL assessment was performed at the following 6 times: before CIRT (baseline), immediately after treatment finished, and at 3 months, 6 months, 1 year and 2 years after CIRT. The assessment was conducted in various ways, including face-to-face interviews, letters and online questionnaires; however, every patient had at least one face-to-face interview QOL assessment by an interviewer with a psychology background. The castration conditions were asked at each follow-up.

### Statistical analysis

SPSS version 23.0 software was used. Nonparametric test and chi-square test were performed to assess patient characteristics and QOL scores between two prescribed dose groups. First, generalized estimating equations [18] were used to evaluate how QOL changed over time compared with baseline. 1/2 standard deviation (SD) from the baseline score, defined as the minimally important change [19], was also used in the quality-of-life. Then, if the QOL score at the last follow-up was lower than the baseline score, we thought the quality-of-life was declined. Next, Wilcoxon’s test and Fisher’s exact test were used for the univariate analysis, and a multivariable logistic regression was performed to correlate clinical parameters with occurrence of acute GU toxicity and reduced QOL. Variables that were statistically significant on univariate analysis (*P* < .10) were then analyzed in a multivariate regression model [20, 21]. *P* values less than.05 were considered significant. Age was considered as a confounding factor [22].

## Results

### Patient characteristics

Table 1 shows the characteristics of the total 64 patients. There was no significant difference in baseline characteristics between two groups. All the patients received hormonal therapy except for 3 low-risk patients. Fifty-seven (89.1%) patients had a senior high school education or above, implying that they could provide a clear and precise description of their perceptions. Forty-six (71.9%) men were treated with 59.2–60.8 GyE/16 fx, whereas 18 men (28.1%) received a prescription dose of 66 GyE/24 fx.

**Table 1:** Patient Characteristics

Variables	59.2–60.8 GyE/16 Fx (*n* = 46)	66 GyE/24 Fx (*n* = 18)	P
Age, median year (IQR)	70.5 (66, 76)	73 (66,77)	0.57
NCCN Stage- No. (%)			0.60
Low risk	3 (6.5)	0 (0)	
Middle risk	16 (34.8)	8 (44.4)	
High risk	19 (41.3)	8 (44.4)	
Very high risk	8 (17.4)	2 (11.1)	
T stage- No. (%)			
1-2a	9 (19.6)	6 (33.3)	0.26
2b-2c	28 (60.9)	11 (61.1)	
3–4	9 (19.6)	1 (5.6)	
PSA vertices -No. (%)			
< 10 ng/ml	18 (39.1)	6 (33.3)	0.88
10–20 ng/ml	13 (28.3)	5 (27.8)	
> 20 ng/ml	15 (32.6)	7 (38.9)	

*IQR* interquartile range, *NCCN* National Comprehensive Cancer Network, *PSA* prostate-specific antigen

Until January 2018, the median follow-up was 19 months (range, 3–33 months). 31 (48.4%) patients were not castrated at last follow-up and the reasons were low risk, completed hormonal treatment and intolerable drug toxicity.

### Quality of life and genitourinary/gastrointestinal toxicity

**Fig. 1:**
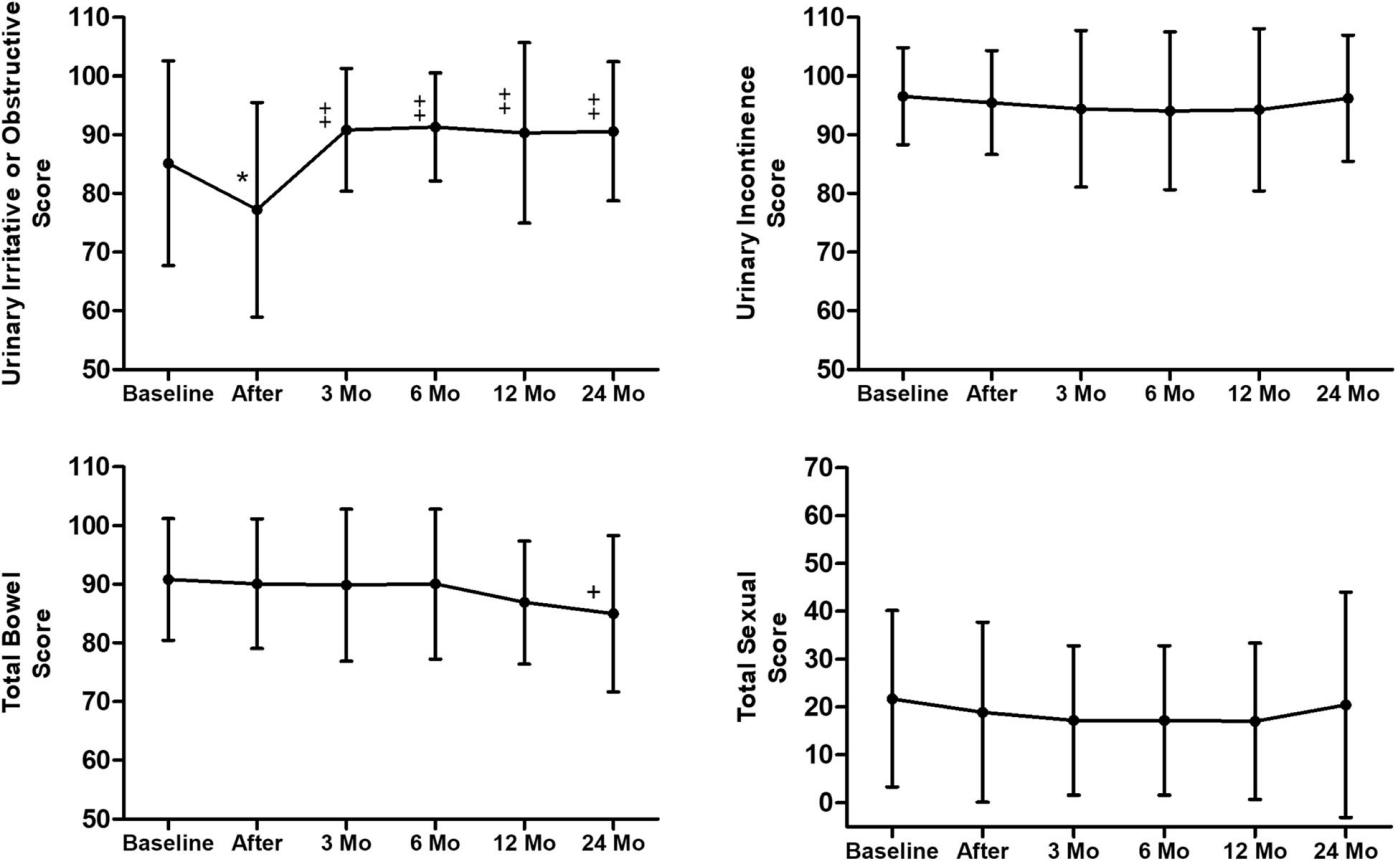
Changes in Quality of Life after Carbon ion Treatment of Prostate Cancer. The graphs show unadjusted changes in mean QOL scores over time for each domain, as measured by the EPIC-26. The error bars represent standard deviations. The scores are on a 0 to 100 scale, with higher values meaning a more favorable health-related quality of life. Asterisks (*) designate time point at which scores significantly differed from baseline (*p* < .01, to account for Bonferroni adjustment) and were significantly clinical relevance. Daggers (+) designate time points at which differences were clinically significant but were not statically significant. Double daggers (‡) designate mean scores that showed improvement over pretreatment levels

**Fig. 2:**
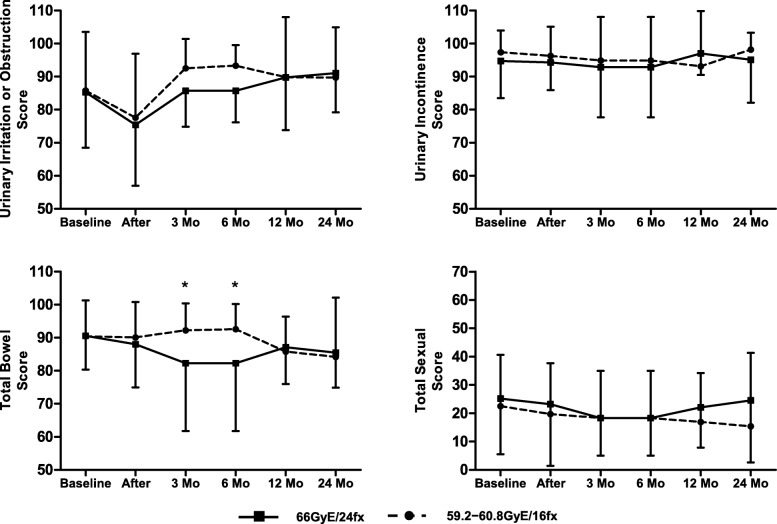
Comparison of Quality of Life after Carbon ion Treatment of Prostate Cancer between two prescribed dose groups. The points and error bars represent mean quality of life scores and standard deviations, respectively. As legend showed, a solid line with square means QOL scores of patients treated with 66 GyE /24 fx, whereas a dashed line with circle means QOL scores of patients treated with 59.2–60.8 GyE /16 fx. Asterisks (*) designate that QOL scores made a significant difference between two prescribed dose groups at some point in time

The questionnaire response rate at each follow-up was over 85% (Table 2). Figure 1 illustrates how QOL changed over time in the domains of urinary irritation or obstruction, urinary incontinence, bowel and sexual function for all patients. The urinary incontinence scores for all patients were unchanged from baseline through 2 years of follow-up at all time points, whereas urinary obstruction/irritation was significantly poorer than baseline at the end of treatment (mean score change ± SD, − 7.92 ± 1.76; *p* < .001) but then recovered or was even superior to baseline in subsequent follow-up. At the end of treatment, when urinary function was at its poorest, patients reported “a moderate or big problem” mostly in urinary frequency (41.4%) and a weak stream (20%); however, these issues subsequently greatly resolved (Table 3). As for bowel function, although two dose groups’ bowel QOL scores differed at 3- and 6-month follow-up, they showed no significant difference in subsequent visit (Fig. 2). For all patients’ bowel QOL, the QOL scores maintained stability in the long term, but at the 2-year follow-up it demonstrated clinically relevant decrease (− 5.83 ± 2.74; *p* = .057). Some 32 (50%) patients had poorer bowel function at last follow-up. Nevertheless, after 6 months of follow-up, the number of patients who reported moderate or large problems with bowel function was zero. When it came to sexual QOL, the scores remained persistently stable after CIRT, but the baseline score was low (21.7 ± 18.5), with over 70% of patients complaining of multiple poor sexual functions (Table 3).

**Table 2:** Patient response rates

	Baseline	After treatment	3 Mo follow-up	6 Mo follow-up	12 Mo follow-up	24 Mo follow-up
No. of patients	64	64	64	57	46	24
No. of patient responses	64	64	55	55	45	22
Response rate, %	100	100	85.9	96.5	97.8	91.7

**Table 3:** Number and percentage of patients reporting specific levels of distress or dysfunction for quality of life domain during follow-up

Quality-of-Life Domain and EPIC Questionnaire Item-No. (%)	Baseline	After	3 Mo	6 Mo	12 Mo	24 Mo
Urinary Irritation or obstruction ^a^						
Dysuria	4 (5.7)	6 (8.6)	0 (0)	0 (0)	0 (0)	0 (0)
Hematuria	5 (7.1)	4 (5.7)	0 (0)	0 (0)	2 (4.4)	0 (0)
Weak stream	9 (12.9)	14 (20)	1 (1.8)	0 (0)	1 (2.2)	1 (4.5)
Frequency	14 (20)	29 (41.4)	3 (5.5)	1 (1.8)	3 (6.7)	1 (4.5)
Overall urinary problem ^a^	12 (17.1)	14 (20)	1 (1.8)	0 (0)	2 (4.4)	1 (4.5)
Bowel function ^a^						
Urgency	4 (5.7)	3 (4.3)	0 (0)	0 (0)	0 (0)	0 (0)
Frequency	5 (7.1)	7 (10)	1 (1.8)	0 (0)	0 (0)	0 (0)
Fecal incontinence	5 (7.1)	4 (5.7)	1 (1.8)	0 (0)	0 (0)	0 (0)
Bloody stools	4 (5.7)	6 (8.6)	1 (1.8)	0 (0)	0 (0)	0 (0)
Rectal pain	4 (5.7)	6 (8.6)	1 (1.8)	0 (0)	0 (0)	0 (0)
Overall bowel problem ^a^	6 (8.6)	8 (11.4)	1 (1.8)	1 (1.8)	1 (2.2)	0 (0)
Sexual function						
Poor erections	49 (70)	48 (68.6)	34 (61.8)	31 (56.4)	34 (75.6)	15 (68.2)
Difficulty with orgasm	53 (75.7)	51 (72.9)	35 (63.6)	31 (56.4)	35 (77.8)	15 (68.2)
Erections not firm	56 (80)	55 (78.6)	37 (67.3)	31 (56.4)	35 (77.8)	16 (72.7)
Erections not reliable	53 (75.7)	50 (71.4)	36 (65.5)	31 (56.4)	35 (77.8)	17 (77.3)
Poor sexual function	54 (77.1)	52 (74.3)	34 (61.8)	29 (52.7)	32 (71.1)	16 (72.7)
Overall sexuality problem ^a^	36 (51.4)	36 (51.4)	20 (36.4)	19 (34.5)	18 (40)	10 (45.5)
Quality of life scores lower than baseline at last follow-up- No. (%)						
Urinary irritation or obstruction	12 (18.8)					
Urinary incontinence	11 (17.2)					
Bowel function	32 (50)					
Sexual function	17 (26.6)					

^a^ Response for these items were bisected on the basis of the response that the QOL distress was "a moderate or big problem"

The occurrences of acute Grade 1 and 2 GU toxicity were 13 (20.3%) and 7 (10.9%), respectively, and the most common GU toxicity was urinary frequency (Grade 1 and 2: 8 and 7, respectively). There were also 5 cases of Grade 1 hematuria and 3 cases of Grade 1 dysuria. None of the patients developed grade 3 or higher acute GU toxicity. There were no acute GI toxicity occurring. With regard to late toxicity, there were 2 (3.1%) cases of Grade 1 and 1 (1.6%) case of Grade 2 cystitis for GU toxicity, with no late GI toxicity observed.

### Clinical factors associated with quality-of-life and acute GU toxicity

Table 4 shows the clinical factors that might affect the QOL after CIRT. TURP was a risk factor that predicted a decline in urinary QOL. Patients with a history of TURP had a 5.7-fold higher probability of poorer irritation/obstruction QOL and a 14.7-fold higher probability of impaired urinary incontinence QOL. Acute GU toxicity, IPSS ≥8 and castration status had no significant association with the urinary QOL decline after CIRT.

**Table 4:** Impact of clinical variables on urinary, bowel and sexual quality-of-life as well as acute genitourinary toxicity

Domain	Covariate	Declined QOL/Toxicity Occurrence ^a^		P	OR ^d^	95%CI ^d^	Adjusted P
		Yes	No				
Irritation/obstruction	Age	69.5 (65, 75.5)	71 (66, 76.5)	0.535	0.954	0.874–1.053	1.043
	Baseline score	92.9 (85.7, 98.2)	89.3 (80.3, 96.4)	0.275	–	–	–
	TURP	33.3%	9.6%	0.036	5.699	1.157–28.08	0.032
	Prescribed dose (59.2–60.8 GyE/16 Fx)	75.0%	71.2%	0.999	–	–	–
	Acute GU toxicity	25.0%	38.5%	0.73	–	–	–
	IPSS ≥8	50.0%	50.0%	0.884	–	–	–
	Castration ^b^	66.7%	48.1%	0.742	–	–	–
Incontinence	Age	69 (67, 76)	71 (66, 76)	0.669	1.000	0.908–1.101	0.997
	Baseline score	100 (91.8, 100)	100 (100, 100)	0.552	–	–	–
	TURP	45.5%	7.5%	0.002	10.214	2.064–50.54	0.004
	Prescribed dose (59.2–60.8 GyE/16 Fx)	63.6%	73.6%	0.475	–	–	–
	Acute GU toxicity	45.5%	34.0%	0.339	–	–	–
	IPSS ≥8	72.7%	45.3%	0.179	–	–	–
	Castration ^b^	45.5%	52.8%	0.296	–	–	–
Bowel	Age	69.5 (65,75.5)	71.5 (67,77)	0.166	0.914	0.840–0.994	0.035
	Baseline score	96.4 (90.2, 100)	89.3 (81.2, 98.2)	0.018	1.102	1.025–1.184	0.008
	Prescribed dose (59.2–60.8 GyE/16 Fx)	71.9%	71.9%	1.000	–	–	–
	Aspirin therapy	15.6%	6.3%	0.426	–	–	–
	Hemorrhoids	25.0%	12.5%	0.337	–	–	–
	Castration ^b^	62.5%	40.6%	0.08	2.799	0.889–8.813	0.079
Sexual	Age	69 (66, 74)	71 (66, 76)	0.726	0.924	0.816–1.046	0.212
	Baseline score	32.7 (26.9, 49.4)	20.1 (2.2, 29.8)	0.007	1.087	1.032–1.144	0.001
	TURP	23.5%	11.8%	0.152	–	–	–
	Prescribed dose (59.2–60.8 GyE/16 Fx)	76.5%	58.8%	0.337	–	–	–
	Castration ^b^	64.7%	41.2%	0.054	13.73	1.987–94.96	0.008
Acute GU toxicity	Age	69 (64, 75)	71 (66, 77)	0.255	0.950	0.875–1.032	0.226
	TURP	14.3%	14.0%	0.972	–	–	–
	Prescribed dose (59.2–60.8 GyE/16 Fx)	57.1%	79.1%	0.069	3.172	0.894–11.26	0.074
	IPSS ≥8	76.2%	37.2%	0.004	5.485	1.601–18.79	0.007
	Castration ^c^	95.2%	95.3%	0.984	–	–	–

^a^ In the column are the percentage of patients or median values (IQR) having the corresponding outcomes of declined quality-of-life or acute toxicity

^b^ Maintaining castration till last follow-up

^c^ Castrated refers to the patients receiving hormone therapy during carbon ion irradiation therapy

^d^ These values were after multivariable adjustment

*QOL* quality of life, *TURP* transurethral resection of the prostate, *GU* genitourinary, *IPSS* international prostate symptom score

The history of TURP did not make a difference to the acute GU toxicity during the CIRT, though it was a remarkable risk factor of declined urinary QOL in the relative long term. Hormonal therapy made no difference, either. However, IPSS ≥8 made a significant difference and it predicted a 5.3-fold higher probability of acute GU toxicity during CIRT (Table 4).

When focusing on bowel QOL, the baseline score was a significant influence factor, with a higher baseline score tending to indicate a decrease in subsequent QOL. Though the discrepancy was not significant in the univariate analysis, it was significant in the multivariate analysis (*p* = .035). Castration status was a potential risk factor for declined bowel QOL with an odds ratio (OR) of 2.8, but it did not reach significance after multifactorial adjustment (*p* = .079). Neither aspirin therapy nor hemorrhoid history were significantly related to impaired bowel QOL.

As for sexual QOL, castration status was a significant risk factor. Patients maintaining castration until the last follow-up had a 12.7-fold increased risk of impaired sexual QOL after adjustments for age and baseline score.

All domains showed no significant difference between the two prescribed doses.

## Discussion

Carbon-ion radiotherapy is a cutting-edge radiation technique; however, fewer than 10,000 patients with prostate cancer worldwide receive CIRT [23]. To date, only few studies had been published about QOL after CIRT for prostate cancer.

Maruyama et al. evaluated the QOL change after 5 years of follow-up in 417 patients with localized prostate cancer who received 66 (63) GyE/20 fx CIRT at the National Institute of Radiological Sciences, using the Functional Assessment of Cancer Therapy (FACT) Prostate scale. Prostate cancer-related QOL scores (FACT-P) decreased immediately after CIRT, but returned to near baseline at 12 months. The global QOL scores (FACT-G) continued to decline slowly. At 5 years of follow-up, the FACT-P and FACT-G scores were significantly lower than the baseline level, but the deviation was only about 2 points. The main reason for the decline in scores was the decline in Social/Family well-being scores.

Ishikawa et al. [24] used the SF-8 scale to study 1-year QOL changes of 76 patients with prostate cancer who received 57.6 GyE/16 fx carbon ion irradiation at the Gumma University Heavy Ion Medical Center. The total Physical Component Summary (PCS) and Mental Component Summary score did not change significantly within 3 months after CIRT; at 1 year, however, the PCS decreased significantly, which was presumably caused by hormonal therapy.

Habl et al. [5] used EORTC PR-55 scale to study the acute adverse reactions and 6-month QOL changes of 92 patients who received 66 GyE/20 fx CIRT (46) or proton therapy (46) for prostate cancer. Scores for urinary symptoms, pain and fatigue all decreased significantly at the end of radiotherapy. Urinary symptoms and pain returned to baseline at 6 months, whereas fatigue remained at poorer levels. Sexually active patients were not affected in terms of sexual function. Patients treated with carbon ions had a better QOL than those treated with protons in both the urinary and gastrointestinal domains.

To summarize the QOL after CIRT of prostate cancer: 1) At the end of CIRT, there was a significant decline in QOL, but it soon recovered. In general, CIRT had little impact on the QOL. 2) All 3 studies found that hormonal therapy was associated with a late-stage decline in QOL scores. The 2 Japanese studies used QOL scales based on global QOL without prostate cancer-related symptoms. The German study found that urinary symptoms were the most affected, but the sample size was small and the follow-up time was short. Above all, differences in QOL scales make it difficult to compare their results. Although our study used the EPIC-26 scale, which is different from the above studies, the results were highly consistent with that of the Heidelberg Ion-Beam Therapy Center, which had indicated that CIRT had little impact on the QOL of patients with prostate cancer. The results of our study are based on the symptoms associated with prostate cancer treatment and will help clinicians better explain the management of symptoms after CIRT for prostate cancer.

In addition to QOL changes, we showed results for acute and late radiation toxicity. The short-term acute toxicity was similar to the results reported in Japan and Germany, where Grade 1–2 acute toxicity was well tolerated and Grade 3+ acute toxicity was not observed [5, 25, 26]. Late radiation toxicity illustrated a low rate of GU toxicity and zero GI toxicity, indicating CIRT in SPHIC could be achieved safely for patients with prostate cancer.

Several studies have proposed that TURP was a predictor of decline in urinary QOL and GU morbidity in photon external radiotherapy [27–29]. This suggestion is based on the fact that TURP induces long-term structural damage to the urethra in the prostate, and photon radiation might have additive effects on the lesions [28, 30]. In patients treated with CIRT, TURP was also a risk factor for poorer urinary QOL. Although the urinary QOL after CIRT was shown to be superior over time, patients with pretreatment TURP needs to be carefully observed.

Advanced age was a significant protective factor against poorer QOL. A previous review by Blank and Bellizzi observed that older cancer survivors when diagnosed had a higher QOL, given that earlier cancer detection can lead to increased stress and an overall negative psychological perception [31]. Nevertheless, many studies have come to the opposite conclusion that age predicted a decline QOL, due to aging-related fatigue, constipation and poorer appetite [22, 32]. This could also be the reason why, in our study, bowel QOL gradually declined as time progressed. From an objective perspective, both statements make sense; thus, age is a relevant but controversial factor in the area of QOL. And we also found that castration status was an underlying predictor of poorer bowel QOL, with an OR of approximately 2.8, though the prediction was on the edge of significance. As not frequently reported, previous studies suggested androgen deprivation therapy (ADT) users had worsening bowel QOL over time compared with non-ADT users who showed improvement over time [33, 34].

With respect to the sexual domain, castration status was a remarkable adverse factor, increasing the risk of impaired sexual QOL by approximately 13-fold. The adverse sexual effects of hormonal therapy have been well investigated, including decreased sexual desire and inability to achieve orgasm [35, 36]. However, according to the EPIC sexual QOL norms in Chinese population with mean age of 69 years, sexual domain scores varied from 22.7 to 27.7 [17, 37]. This might be due to the aging-related decrease in sex hormones, that the elderly become less sexually active, both physically and psychologically. In our study, the sexual QOL scores were approximately 21, and no significant difference was shown before and after CIRT. It has been suggested the influence on sexual QOL of CIRT itself might be minimal, less so than that of aging.

Ishikawa, H. et al. reported that acute GU toxicity was a risk factor for the occurrence of persistent toxicity after CIRT [25]. Therefore, it is necessary to investigate the risk factors for acute toxicity. Among the clinical factors, IPSS ≥8 increased the risk of acute GU toxicity after CIRT. It has been reported that a preradiotherapy IPSS ≥15 is associated with a higher incidence of Grade ≥ 2 acute GU toxicity [38]. The baseline IPSS threshold with acute GU toxicity was lower in CIRT, probably indicating that patients with poor urinary function were more sensitive to acute GU toxicity than those undergoing photon radiotherapy; however, whether it affects long-term toxicity remains to be determined.

Our study has several limitations. First, patients were not followed up long enough to observe enough clinical endpoint events; thus, the late toxicity of CIRT needs to be further updated in subsequent reports from our center. Furthermore, the number of the patients in this study was not as large as that using photon radiotherapy, possibly resulting in selection bias. However, as far as 2016, the number of all carcinoma patients receiving CIRT per year was 938, and only 5 of all 11 heavy ion centers admitted over 1000 patients, with 4 facilities located in Japan and 1 in Germany [39].

### Conclusions

Patients with prostate cancer had favorable QOL after CIRT. IPSS ≥8 was a risk factor for acute GU toxicity, and TURP predicted a decline in urinary QOL. Age was related to bowel QOL and castration status was associated with sexual QOL.

## Data Availability

The datasets used and/or analyzed during the current study are available from the corresponding author on reasonable request.

## Abbreviations

ADT: Androgen deprivation therapy
CIRT: Carbon ion radiotherapy
CTV: Clinical target volume
EORTC: European Organisation for Research and Treatment of Cancer
EPIC-26: 26-item version of Expanded Prostate Cancer Index-Composite
FACT: Functional Assessment of Cancer Therapy
GI: Gastrointestinal
GU: Genitourinary
HIT: Heidelberg Ion-Beam Therapy Center
IPSS: International prostate symptom score
IQR: Interquartile range
NCCN: National Comprehensive Cancer Network
PCS: Physical Component Summary
PSA: Prostate-specific antigen
QOL: Quality-of-life
RTOG: Radiation Therapy Oncology Group
SD: Standard deviation
SPHIC: Shanghai Proton and Heavy Ion Center
TURP: Transurethral resection of prostate
